# Implementation of a gait center training to improve walking ability and vital parameters in inpatient neurological rehabilitation- a cohort study

**DOI:** 10.1186/s12984-020-00669-3

**Published:** 2020-03-04

**Authors:** Stephanie Reichl, Franz Weilbach, Jan Mehrholz

**Affiliations:** 1 Klinik Bavaria Bad Kissingen, Von-der-Tann-Straße 18- 22, 97688 Bad Kissingen, Germany; 2 Department of Public Health, Dresden Medical School, Technical University Dresden, 01062 Dresden, Germany; 3 Wissenschaftliches Institut, Private Europäische Medizinische Akademie der Klinik Bavaria in Kreischa GmbH, An der Wolfsschlucht 1-2, 01731 Kreischa, Germany

**Keywords:** Stroke, CIP/CIM, Rehabilitation, Gait center training, Walking, Vital parameter

## Abstract

**Background:**

Many studies showed that robot-assisted gait training might improve walking of patients after stroke. The question remains whether patients with other neurological diagnoses can improve their ability to walk by training in a gait center. Aim of the present study was therefore to investigate the effects of a gait center training in inpatient neurological rehabilitation on walking ability.

**Methods:**

We implemented a gait center training in addition to individual inpatient rehabilitation. Our primary outcome was walking ability based on the Functional Ambulation Categories (FAC). Our secondary outcomes were vital capacity and blood pressure. We predefined subgroups of patients with ischemic and hemorrhagic stroke and critical illness myopathy (CIM) and polyneuropathy (CIP).

**Results:**

We included 780 patients from our inpatient rehabilitation center in our cohort study. We analyzed 329 patients with ischemic, 131 patients with hemorrhagic stroke and 74 patients with CIP/ CIM.

A large number of patients were able to improve their ability to walk. At the end of rehabilitation, patients with ischemic stroke and FAC 3 = increased theirFAC scores by 5%, FAC 4 = 4% and FAC 5 = 7%. Patients with hemorrhagic stroke and FAC 3 = increased by 5%, FAC 4 = 11% and FAC 5 = 9% and patients with CIP/CIM increased by FAC 3 = 3%, FAC 4 = 22% and FAC 5 = 26%.

The largest improvement in walking ability during rehabilitation had patients with a FAC = 1 at baseline who improved by a median of 1.4 FAC points (*p* < 0.001). After adjusting for the number of gait training sessions, the largest improvement in walking ability during rehabilitation had patients with a FAC = 0 at baseline who improved by 1.8 FAC points (*p* < 0.001).

**Conclusions:**

Implementation of an additional gait center training may significantly improve walking ability in neurological rehabilitation.

## Background

Improvement of walking ability is a major goal in neurological rehabilitation [1]. It has been described that moderately intense physical activity in healthy adults is about 100 steps per minute. Patients with stroke, however, manage only one third of this, due to disease-related immobilization [2], and might therefore have reduced cardiopulmonary endurance [3, 4]. Therefore, many patients are usually only able to engage in moderately intensive physical activity for about 150 min per week [5]. Furthermore, patients with a neurological disorder such as a stroke have an increased risk of developing cardiovascular disease, a reinfarction, a heart attack or other heart problems due to such deconditioning and immobilization [2].

Gait disorders are one of the most common complaints in up to 60% of patients with cerebrovascular diseases [6]; and up to 80% of patients after stroke being affected [7]. It has been shown that aerobic capacity correlates with gait recovery [8, 9] and it is well known, that patients after stroke are limited in their activities of daily living such as walking due to reduced aerobic capacity [4]. It has been described that the cardiovascular fitness of patients after a stroke is no more than 50% of the performance level of healthy subjects [3]. Therefore improvement of cardiopulmonary fitness seems to be a prerequisite for good functional outcome and for the prevention of cardio-respiratory complications [10]. In order to reach such recommended level of physical activity, training in a gait center is used in neurological rehabilitation in addition to conventional gait rehabilitation. A gait center training consists of the elements standing and balancing trainer, robot-assisted gait training system and a treadmill with safety belt. With these devices, it is possible for patients to be mobilised vertically at an early stage, depending on their functional capacity and cardiopulmonary endurance [11]. Patients, who used the robot-assisted gait training, were able to walk of up to 1000 steps [12]. For instance many studies demonstrated the successful use of robot-assisted gait training to increase physical activity of inpatient stroke patients [1, 13, 14]. Patients after stroke who are, however, unable to walk at the beginning of rehabilitation, defined as a Functional Ambulation Categories Score (FAC) of 0 to 2, benefited most from robot-assisted gait training in terms of walking ability [1].

Until now it is not clear whether patients with other neurological diagnoses would improve their walking ability by training in a gait center in a similar way as patients after a stroke. Besides the development of walking ability, other questions seem to be important. For example, it is not yet clear whether the initial walking disability or the diagnosis influences the improvement of vital parameters by robot-assisted gait training in a gait center. Currently, only preliminary data are available on the use of treadmill training for patients who are unable to walk [15]. What we know is that neurological diseases may have an impact on static pulmonary volume and expiratory capacity [16]. Reduced lung ventilation leads to a reduced level of physical activity and increases the risk of broncho-pulmonary complications in neurological patients [17] and immobilised patients [18, 19]. The maximum energy consumption of neurological patients averages 3.77 metabolic equivalents (MET) [20], while healthy people have between 8 to 10 MET [4]. An average MET of 3.77 seems, however, not sufficient to walk with a normal gait speed [20, 21]. There is evidence that robot-assisted gait training might also improve cardiopulmonary endurance [8].

Daily training in a gait center in inpatient rehabilitation could therefore be useful in addition to early intensive mobilization for patients in neurological rehabilitation. In our view, however, it is time to investigate the effects of a gait training center training on walking ability, cardiopulmonary outcomes with a larger sample of patients.

The aim of the present study was, therefore, to investigate the effects of additional training in a gait center training on walking ability and vital capacity and blood pressure during inpatient neurological rehabilitation. Our main hypothesis was that walking ability and vital parameters improve.

## Methods

### Protocol and registration

We published a study protocol, which has been registered in the DRKS database under the ID DRKS00014090.

### Design

This is a cohort study of patients being in their inpatient neurological rehabilitation.

### Participants

We recruited all patients of the ‘Klinik Bavaria’ in Bad Kissingen, our neurological rehabilitation department, between July 2015 and June 2016 according to the following inclusion and exclusion criteria. Inclusion criteria were the ability to participate in the training program of our gait center.

Exclusion criteria were: 1) no gait training in gait center due to deterioration of general condition, a referral to a primary care clinic, isolation due to multi resistant germs and not able to be mobilised into a wheelchair and 2) contraindications to treatment with robot-assisted gait training system such as implantation of a cardiac pacemaker, artificial joint replacement in hip and knee joints up to two years after implantation, osteoporosis, manifested leg length difference or scoliosis or psychotic illnesses. The maximum weight limit of subjects was 150 kg.

### Setting

All patients underwent their individual inpatient rehabilitation, including daily physiotherapy (e.g. lower leg strength, balance and gait training over ground), occupational therapy (e.g. hand-arm training) and speech therapy. As an add-on therapy patients received gait training center training for up to five days per week during their entire rehabilitation stay. The additional sessions in the gait center lasted up to 30 min. As a main requirement for treatment in our gait center patients had to be able to sit in alone in a wheelchair.

### Intervention

The therapeutic framework of our gait center, which consists of the elements standing and balancing trainers, robot-assisted gait training system and a treadmill with safety belt [11] has been steadily gaining acceptance in neurological rehabilitation. Therapy with these different devices enables patients to have social interaction and it also might increase their motivation to exercise since all these types of devices are available for each patient and they are able to train at the same time with other patients [11, 12].

According to the functional abilities at the beginning of rehabilitation, measured by means of Functional Ambulation Categories (FAC) [22–24], the patients trained with the most appropriate element in our gait center. E.g. patients with a FAC of 0 started their training in the gait center with the standing and balancing trainer. If patients with a FAC 0 to 2 were able to cope for 15 min in the standing and balancing trainer, they were trained with a robotic gait trainer [11].

The robot-assisted gait training in our gait center uses the end effector technology with two G-EO System Evolution and one G-EO System Basic (Reha Technology, Olten, Switzerland). The amount of activity on the robot-assisted gait training system varied between 50 to 1000 steps per session [25]. The therapeutic options for treatment in the robot-assisted gait training system included walking training in passive (G-EO System Basic), active assistive and active modes, and climbing stairs (G-EO System Evolution). The robot-assisted gait training system enables the regaining of motor coordination in a safe, intensive, task-specific and repetitive setting [10]. Therefore, this training setting is especially suitable for patients who are unable to walk. Participants in the gait center training sessions also performed standing balance training and treadmill training during the inpatient rehabilitation period and not just robotic gait training. E.g. if patients were unable to train with the robot-assisted gait system due to cardiopulmonary instability or reduced vigilance and trunk control, an alternative intervention was conducted using standing and balancing trainers (Thera Trainer, Hochdorf, Germany).

In this study, however, we analysed the number of gait training sessions through robotic-assisted gait training only.

### Clinical assessments

We used the Functional Ambulation Categories (FAC) as the primary outcome to measure walking ability from 0 (not able to walk) to 5 (independent walking ability) [22–24].

We used vital capacity (VC) as the secondary outcome, which was defined as the air volume of an exhalation (expiratory vital capacity EVC) [26]. We then recorded the volume between maximum inhalation and exhalation. We measured that volume by a pocket spirometer (spirotest, Riester, Jungingen) of the dry spirometer type [14], and recorded the EVC in ccm. The pocket spirometer records an air volume of 1000 to 7000ccm, divided into distances of 100ccm. The readings of turbine spirometers were assumed to be about 5 % below the exact value [16].

We used blood pressure (BP), as a secondary outcome which was measured daily by inpatient nursing staff with blood pressure cuffs (clinicus II- boso, Jungingen, Germany) and stethoscope manually indirectly to Riva-Rocci.

In patients with continuous monitoring, blood pressure was measured automatically (Infinity® Gamma XL- Dräger, Lübeck, Germany; BSM-2301 K- NIHON KODEN, Rosbach v.d.H., Germany) and in patients without nursing care independently (medicus uno- boso, Jungingen, Germany) in mmHg. The daily recorded systolic and diastolic blood pressure readings were averaged over weekly averages for each week of rehabilitation stay per patient. We compared the values of all outcome measurements at the beginning of rehabilitation and at the end of rehabilitation in the context of this study.

### Statistical analysis

We adhered to the SAMPL guidelines for statistical analysis [27]. As part of the descriptive data analysis, we calculated the measure of dispersion for the entire population as well as for the three selected subgroups. We considered the outcome data at the beginning of the rehabilitation as well as at the end of the rehabilitation. We assumed that there was a clear spread of the evaluated subgroup since no patient exclusion was based on age, disease duration or other characteristic features due to the study design. Therefore, we used non-parametric tests because we could not assume a normal distribution. We used medians with corresponding interquartile range (IQR) for plotting the development of the groups. We evaluated the steady dependent variables through paired Wilcoxon tests. In order to assess the improvement in walking ability, we calculated in a first analysis the gain in the FAC score as follows:

*FAC change* = (FAC score at end of inpatient rehabilitation) minus (FAC score at the start of inpatient rehabilitation).

In a second analysis we calculated an Analysis of Covariance (ANCOVA) and adjusted for the individual number of gait therapy sessions experienced by participantsusing the number of gait training sessions as co-variate and computed least squares mean estimates for the FAC change, with 95% Confidence interval for the adjusted mean FAC change (95%CIs for the least squared mean estimates),

The significance level was defined as alpha = 5% and we adjusted for alpha inflation with Bonferroni correction. All statistical analysis was done with the software R 3.3.2 and SAS 9.4 (SAS Institute Inc., Cary, NC, USA).

## Results

### Patient characteristics

**Fig. 1:**
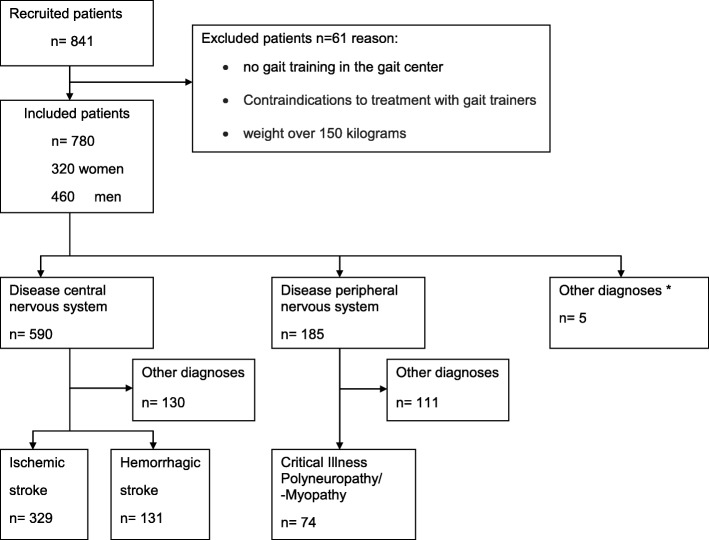
Flow chart- Patient recruitment and diagnosis distribution. (* cancer and tumors)

We included 780 patients, among them 590 patients with central nervous system diseases, 185 patients with a disease of peripheral system and 5 patients with other diagnoses (cancer and tumors) (see Fig. 1, Flow chart).

We analyzed 329 patients with ischemic stroke (STI), 131 patients with hemorrhagic stroke (STH) and 74 patients with critical illness polyneuropathy or critical illness myopathy (CIPM). We found differences in patient characteristics between patients with central and peripheral neurological diagnoses. Patients with STH and STI received gait center therapy after a shorter period of illness (3 to 4 weeks) compared to other neurological diagnoses and the duration of rehabilitation was shorter (5 weeks). Patients with a peripheral diagnosis (CIPM) received gait center therapy at a later disease duration (9 weeks) and took longer to improve (6 weeks, as shown in Table 1).

**Table 1:** Patient characteristics at baseline from total sample and selected subgroups

	Total sample (*n* = 780)		Central Diagnoses							Peripheral Diagnoses			NWA			*p* value
			STI (*n* = 329)			STH (*n* = 131)				CIPM (*n* = 74)			(*n* = 89)			
	Median	IQR	n	Median	IQR	n	Median	IQR	n	Median	IQR	n	Median	IQR	n	
Age (years)	68.0	22.0	780	72.0	18.0	329	72.0	23.0	131	71.0	14.8	74	72.0	8.0	89	0.496
duration of illness (weeks)	4.3	16.6	629	3.0	5.6	284	3.9	10.8	109	9.0	10.5	51	3.7	6.7	73	< 0.001
duration of rehabilitation (weeks)	4.9	3.9	780	4.6	3.0	329	5.4	4.2	131	6.3	4.3	74	6.0	4.6	89	< 0.001
time rehabilitation beginning-start gait trainer (weeks)	0.7	1.0	616	0.7	0.9	266	0.7	1.5	102	1.7	3.5	43	3.4	5.7	26	< 0.001
total gait trainer- sessions	5.0	10.0	779	5.0	9.0	329	7.0	13.5	131	1.0	8.5	74	1.0	1.3	88	< 0.001
FAC	3	5	750	3	5	314	1	4	126	0	2	73	0	0	89	< 0.001
Vital capacity (ccm)	2100	1500	594	2100	1400	253	1900	1525	92	1600	1450	44	1300	800	40	0.052
Blood pressure systolic (mmHg)	129	17	762	130	43	323	129	17	128	124	16	72	129	17	87	< 0.001
Blood pressure diastolic (mmHg)	76	11	762	76	11	323	77	5	128	74	9	71	76	10	87	0.006

*STI* ischemic stroke, *STH* hemorrhagic stroke, *CIPM* Critical Illness Polyneuropathy or –Myopathy, *NWA* patients who did not reach walking ability neither at the beginning nor at the end of rehabilitation- this subgroup *n* = 89 is composed as follows 45(STI) + 24(STH) + 10(CIPM) + 10(other neurological diagnosis)*, IQR* interquartile range, *FAC* Functional Ambulation Categories, *p value-* derived from paired Wilcoxon test

### Walking ability

**Fig. 2:**
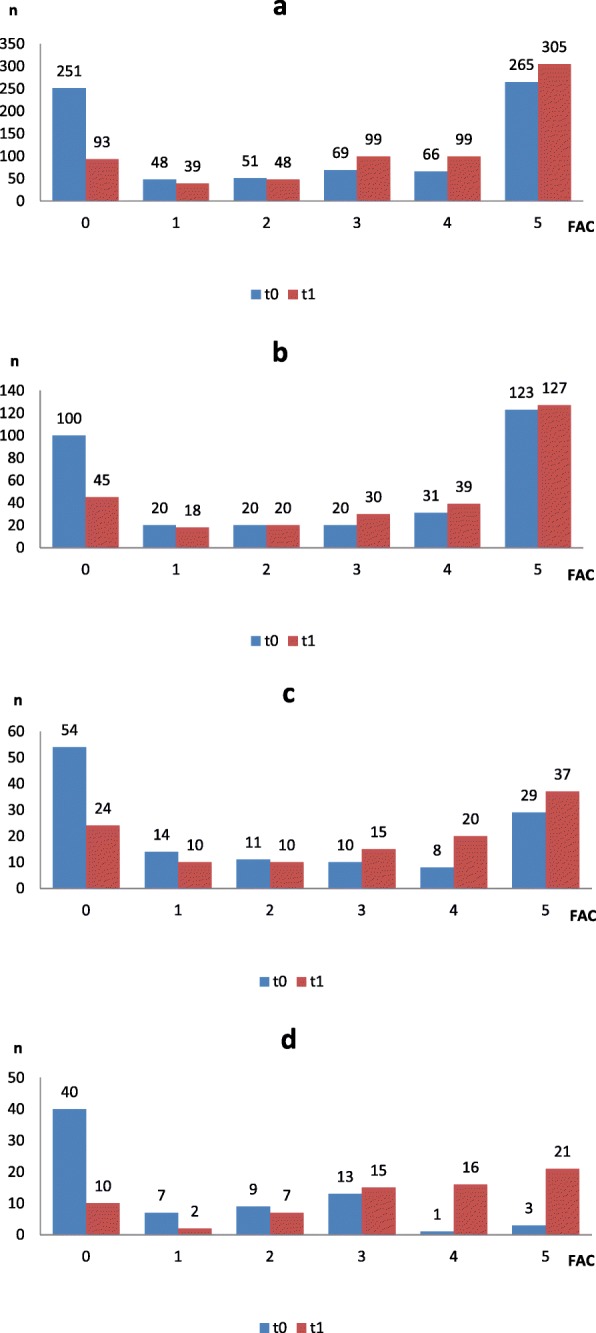
**a-d**. Regaining walking ability in the rehabilitation stay between start of rehabilitation (t0) and end of rehabilitation (t1); differentiated according to FAC score at t0. (**a**- *total sample*, **b**- *STI-* ischemic stroke, **c**- *STH*- hemorrhagic stroke, **d**- *CIPM-* Critical Illness Polyneuropathy or –Myopathy)

The percentage of participants with a walking ability of FAC ≥ 3 increased from 53 to 75% during the rehabilitation process (see Fig. 2a). In the total sample, the largest improvement in walking ability during the rehabilitation process had patients with a FAC = 1 at baseline who improved by 1.4 FAC points (*p* < 0.001) (Table 2). After adjusting for the number of gait training sessions, in the total sample, the largest improvement in walking ability during rehabilitation had patients with a FAC = 0 at baseline who improved by 1.8 FAC points (*p* < 0.001) (Table 3).

**Table 2:** Regaining walking ability, changes in Functional Ambulation Categories

				Central diagnoses						Peripheral diagnoses		
	Total sample (*n* = 780)			STI (*n* = 329)			STH (*n* = 131)			CIPM (*n* = 74)		
	FC	IQR	*p* value	FC	IQR	*p* value	FC	IQR	*p* value	FC	IQR	*p* value
FAC at t0												
0	1.0*	2.0	< 0.001	0.3	1.6	< 0.001	0.4	1.9	< 0.001	1.8*	3.1	< 0.001
1	1.4*	2.4	< 0.001	1.3*	2.2	0.004	1.5*	3.4		2.2*	2.2	0.021
2	1.0*	1.0	< 0.001	1.0*	1.0	0.002	1.5*	1.1	0.006	2.4*	1.8	0.013
3	1.0*	1.7	< 0.001	1.0*	1.3	0.001	1.5*	0.8	0.013	1.3*	0.9	0.002
4	0.4	1.0	< 0.001	0.0	1.0	< 0.001	1.1*	0.8	0.019	0.9	0.0	1.000
5									0.037			

*FAC* Functional Ambulation Categories,

*FC* FAC change- Median Gain in walking ability between t0(start of rehabilitation) and t1(end of inpatient rehabilitation),

*IQR* interquartile range from FC,

*p value*- derived from paired Wilcoxon test- *p < 0.05 and clinically relevant (minimal improvement by one FAC point),

*STI* ischemic stroke,

*STH* hemorrhagic stroke,

*CIPM* Critical Illness Polyneuropathy or -Myopathy

**Table 3:** Regaining walking ability, changes in Functional Ambulation Categories adjusted by the number of gait training sessions

				Central diagnoses						Peripheral diagnoses		
	Total sample (*n* = 780)			STI (*n* = 329)			STH (*n* = 131)			CIPM (*n* = 74)		
	FCadj	95%CI	*p* value	FCadj	95%CI	*p* value	FCadj	95%CI	*p* value	FCadj	95%CI	*p* value
FAC at t0												
0	1.8*	1.7; 2.0	< 0.001	2.1*	1.9; 2.3	< 0.001	1.9*	1.8; 2.1	< 0.001	1.7*	1.5; 1.8	< 0.001
1	1.5*	1.2; 1.9	< 0.001	1.6*	1.2; 2.1	< 0.001	1.6*	1.2; 2.0	< 0.001	1.4*	1.0; 1.7	< 0.001
2	1.2*	0.9; 1.5	< 0.001	1.4*	0.9; 1.8	< 0.001	1.2*	0.8; 1.5	< 0.001	1.0*	0.7; 1.3	< 0.001
3	0.9	0.6; 1.2	< 0.001	0.8	0.5; 1.2	< 0.001	0.9	0.6; 1.2	< 0.001	0.8	0.5; 1.1	< 0.001
4	0.5	0.2; 0.8	< 0.001	0.5	0.1; 0.9	0.048	0.5	0.2; 0.8	0.013	0.5	0.2; 0.8	0.006
5												

*FAC-* Functional Ambulation Categories,

*FCadj* FAC change adjusted by the number of gait training sessions received – adjusted mean gain in walking ability between t0(start of rehabilitation) and t1(end of inpatient rehabilitation) revealed by Analysis of Covariance (ANCOVA) using number of gait training sessions as co-variate and computation of least squares mean estimates for the classes FAC at t0,

*95%CI* – 95% Confidence interval for the adjusted mean FC (95%CIs for the least squares mean estimates for the classes FAC at t0),

*p value*- derived from ANCOVA least squares estimates for FAC at t0, *p < 0.05 and clinically relevant (minimal improvement by one FAC point),

*STI* ischemic stroke,

*STH* hemorrhagic stroke,

*CIPM* Critical Illness Polyneuropathy or -Myopathy

Improvements of the ability to walk differed between patients with central and peripheral diagnosis (Tables 2 and 3).

In the subgroup STI, the percentage of participants with a walking ability of FAC ≥ 3 increased from 55 to 71% during the rehabilitation process (see Fig. 2b). The largest improvement in walking ability during rehabilitation had patients with a baseline FAC of 1 who improved by 1.3 FAC points (*p* = 0.004) (as shown in Table 2). After adjusting for the number of gait training sessions, the largest improvement in walking ability during rehabilitation, had patients after STI and with a FAC = 0 at baseline who improved by 2.1 FAC points (*p* < 0.001) (as shown in Table 3).

In the subgroup STH, the percentage of participants with a walking ability of FAC ≥ 3 increased from 37 to 62% during rehabilitation (see Fig. 2c). Patients with a baseline FAC score of equal to 1, 2 and 3 had the largest gain of FAC score (p = 0.004, 0.002 and 0.001, respectively; as shown in Table 2).

After adjusting for the number of gait training sessions, the largest improvement in walking ability during rehabilitation, had patients after STH with a FAC = 0 at baseline who improved by 1.9 FAC points (*p* < 0.001) (as shown in Table 3).

Furthermore, in the subgroup CIPM, the percentage of participants with a walking ability of FAC ≥ 3 increased from 23 to 74% during the rehabilitation process (see Fig. 2d). Patients with CIPM and baseline FAC scores of 3 had the largest gain of FAC score by 2.4 FAC points (*p* = 0.013, as shown in Table 2).

After adjusting for the number of gait training sessions, the largest improvement in walking ability during rehabilitation, had patients after CIPM with a FAC = 0 at baseline who improved by 1.7 FAC points (*p* < 0.001) (as shown in Table 3).

In total 89 patients did not reach walking ability, 79 patients from the subgroups (STI = 45, STH = 24, CIPM = 10) and ten patients with other neurological diagnoses (Table 5).

As shown in Fig. 2a-d a number of patients dropped out until the end of rehabilitation, due to medical reasons, transfer back to acute hospital, palliative treatment or early discharge to a nursing home.

### Vital capacity

The mean vital capacity (VC) improved by 300ccm (*p* < 0.001) in the total sample with a baseline FAC of 0. The mean VC of Patients with a baseline FAC of 1 to 5, however, did not improved significantly.

In patients in the STI and STH subgroup, who had a FAC of 0 at baseline, VC improved significantly by 200ccm (*p* = 0.02) and 500ccm (*p* = 0.001), respectively (Table 4).

**Table 4:** Development vital capacity

				Central diagnoses						Peripheral diagnoses					
	Total sample (*n* = 780)			STI (*n* = 329)				STH (*n* = 131)		CIPM (*n* = 74)			NWA (*n* = 89)		
	VC t0	VC t1	*p* value	VC t0	VC t1	*p* value	VC t0	VC t1	*p* value	VC t0	VC t1	*p* value	VC t0	VC t1	*p* value
FAC at t0															
0	1500	1800	< 0.001*	1500	1700	0.022*	1200	1700	0.001*	1900	1800	0.195	1300	2000	0.01*
1	1650	2000	0.079*	1900	2000	0.475*	1450	2400	0.052*	1300	1700	0.500			
2	1500	1500	0.047*	1200	1200	0.291*	1800	2350	0.098*	1300	1300	1.000			
3	1900	2000	0.832*	1600	1650	0.385*	1450	2350	0.219*	2000	2200	0.609			
4	2000	2300	0.028*	2050	2150	0.559*	2200	2200	1.000*	NA	NA	NA			
5	2700	2800	0.062*	2700	2500	0.488*	2700	2850	0.195*	3900	3200	1.000			

*FAC* Functional Ambulation Categories, *VC* Median vital capacity in Cubic centimeter (ccm), *t0*- start of inpatient rehabilitation, *t1*- end of inpatient rehabilitation, *p value*- derived from paired Wilcoxon test- *p < 0.05, *STI* ischemic stroke, *STH* hemorrhagic stroke, *CIPM* Critical Illness Polyneuropathy or –Myopathy, *NWA* patients who did not reach walking ability neither at the beginning nor at the end of rehabilitation- this subgroup *n* = 89 is composed as follows 45(STI) + 24(STH) + 10(CIPM) + 10(other neurological diagnosis)

In patients with STI and STH who regained walking function, and patients with CIPM, however, VC did not improve significantly.

### Blood pressure

The blood pressure, BP, in the total sample was at baseline already within a normal range with a systolic BP of 129 mmHg and diastolic BP of 76 mmHg. No significant improvement of BP during rehabilitation was found (systolic BP of 127 mmHg and diastolic BP of 76 mmHg at the end of rehabilitation, *p* = 0.05 and *p* = 0.38, respectively). There were no differences neither for systolic nor for diastolic BP between the subgroups STI, STH and CIPM during rehabilitation (Table 5).

**Table 5:** Development blood pressure

				Central diagnoses						Peripheral diagnoses					
	Total sample (*n* = 780)			STI (*n* = 329)				STH (*n* = 131)		CIPM (*n* = 74)			NWA (*n* = 89)		
	BP t0	BP t1	*p* value	BP t0	BP t1	*p* value	BP t0	BP t1	*p* value	BP t0	BP t1	*p* value	BP t0	BP t1	*p* value
FAC at t0															
0	128/75	124/75	0.08/0.68	130/74	126/76	0.44/0.32	131/78	124/74	0.04/0.07	123/74	123/73	0.91/0.76	129/76	124/74	0,06/0,01
1	127/75	126/76	0.37/0.56	126/74	128/72	0.42/0.84	127/77	124/77	0.51/0.88	139/77	131/72	0.38/0.07			
2	124/76	127/75	0.43/0.99	133/77	130/75	0.46/0.17	121/74	128/78	0.44/0.24	117/73	127/73	0.04/0.40			
3	128/75	126/75	0.69/0.83	129/75	130/76	0.34/0.22	124/74	121/74	0.95/0.86	131/72	126/76	0.91/0.61			
4	129/78	129/77	0.34/0.87	128/76	127/74	0.07/0.29	134/78	129/80	1.00/0.56	105/64	111/68	1.00/1.00			
5	130/80	130/80	0.10/0.16	133/80	132/79	0.01/0.01	130/80	130/80	0.98/0.43	129/80	121/85	0.50/0.50			

*FAC* Functional Ambulation Categories, *BP* Median blood pressure in millimeters of mercury (mmHg), *t0*- start of inpatient rehabilitation, *t1*- end of inpatient rehabilitation, *p value*- derived from paired Wilcoxon test- *p < 0.05 and clinically relevant, *STI* ischemic stroke, *STH* hemorrhagic stroke, *CIPM* Critical Illness Polyneuropathy or –Myopathy, *NWA* patients who did not reach walking ability neither at the beginning nor at the end of rehabilitation- this subgroup *n* = 89 is composed as follows 45(STI) + 24(STH) + 10(CIPM) + 10(other neurological diagnosis)

## Discussion

The present prospective cohort study is the first study to examine a relatively large sample of 780 patients during inpatient rehabilitation in a gait center. It is one of the first studies in neurological rehabilitation that evaluates the improvement of walking ability by comparing different diagnosis without excluding several comorbidities. The results of this study are therefore be applicable to inpatient neurological rehabilitation.

We claimed that walking ability and vital parameters can be improved with gait center training. In addition, we hypothesized that the training improves or stabilizes the vital parameters of patients who are unable to walk in the rehabilitation process.

As a main result, we found that gait training can improve walking ability and provides the greatest benefit to specific patient subgroups e.g. patients after stroke. Gait center training seems to be most effective for nonambulatory patients with an initial walking ability of FAC equal to 1. After adjusting for the number of walking training sessions, we found that patients with FAC equal to 0 in particular benefit most from gait center therapy.

A recent Cochrane Review suggested recently robot-assisted gait training might be effectivefor non-ambulatory patients (FAC 0 to 2) [1]. Our results are in this line with a recommendation for gait center training especially for patients with an initial FAC Score of 0–2.

A recent network-meta-analysis found that robot-assisted gait training after stroke might be effective if end-effectors are used [12]. Our study supports the argument that even non-ambulatory patients with an FAC of 0 to 2 could benefit and improve their walking ability if end-effector gait training is applied.

We could not confirm our hypothesis that patients who were able to walk in the rehabilitation process would also improve in vital parameters. Our results shows, however, that the vital capacity of those patients who are unable to walk at rehabilitation onset might improve. This effect was consistent in all diagnostic groups. The results are somewhat in contrast to the literature who described that robot-assisted gait and treadmill training has the potential to produce aerobic exercise for patients with ‘limited and incapable disease’ and in patients who are already able to walk [2, 15].

As described in the literature we observed in patients after stroke, impaired breathing due to weakened respiratory muscles and reduced chest mobility when breathing in and out [28]. Especially nonambulatory patients spend much time of the day sitting in a wheelchair which might reinforce this problem. Additional training in the gait centre could interrupt such a vicious circle of physical inactivity, unfavorable immobilized position and reduced lung volume and our results shows effects on VC for patients with FACs equal to 0.

Oxygen demand during walking is greater in patients with ischemic stroke than in healthy controls [29]. In order to meet this demand, a sufficiently large gas exchange is necessary. If this cannot be provided e.g. due to decreased chest mobility and reduced lung volumes, walking distances might be shorter than necessary.

Additional mobilization through gait center training could increase the cardiopulmonary load and thus promote breathing frequency and respiratory effort [30].

### Limitations

This study has some potential limitations. First, this was a single-center study and it needs a reevaluation in a multicenter design. Second, this was just an observational study and no causal relationship can be drawn. This study is therefore more likely to be seen as a feasibility analysis of a relatively large cohort of patients in neurological inpatient rehabilitation.

We analysed to what extent the gait center training can be used appropriately with the available equipment capacities. In this study, we found that in some cases the patients remained well below the target of five training applications. This is due to drop outs such as acute treatment or deterioration of the patients’ general condition. Future studies should therefore aim to reduce the discrepancy between target and effective therapies by developing appropriate prognostic parameters.

The effects on walking ability as well as vital capacity result from a combination of physiotherapy together with all other components from the gait center such as therapies in the standing trainer and on the treadmill, robot-assisted gait training and all other treatment components in an individually adapted rehabilitation. The improvements showed can therefore not only be attributed to the robot-assisted gait therapy, but are also due to a consistent early mobilization into a vertical position. We focused in this study, however, only on the number of robotic-gait training sessions and used this as an important factor when adjusting our results.

Another limitation in this study is that only gait ability, VC and BP was measured. In future studies, activities of daily life, physical activity, walking ability at home and quality of life should also be measured.

It should be considered that the use of a turbine pocket spirometer to measure VC during expiration is less meaningful than the use of a pneumotachograph. Since the measurement is not a purely mechanical determination of the measured values, neither a respiratory curve nor detailed measurement results are recorded digitally. The determination of the coarse value can only be read off visually by the measuring personnel on the spirometer scale at 100 ml intervals.

## Conclusions

The present study showed for the first time in a large cohort study in inpatient neurological rehabilitation the clinical effects of an additional gait center training on walking ability.

## Data Availability

The datasets supporting the conclusions of this article are included within the article, figures and tables.

## Abbreviations

BP: Blood pressure
ccm: Cubic centimeter
CIPM: Critical Illness Polyneuropathy or -Myopathy
EVC: Expiratory vital capacity
FAC: Functional Ambulation Categories
IQR: Interquartile range
MET: Metabolic equivalents
mmHg: Millimeters of mercury
STH: Hemorrhagic stroke
STI: Ischemic stroke
VC: Vital capacity
